# Supplementary material for: Identification of photoperiod-induced specific miRNAs in the adrenal glands of Sunite sheep (Ovis aries)
Source: Front Vet Sci. 2022 Jul 22;9:888207. doi: 10.3389/fvets.2022.888207 (PMC9354845; doi:10.3389/fvets.2022.888207)
Supplement: Supplementary file 1 [file Data_Sheet_1.ZIP › Supplementary file/miRDeep2 file.zip!/miRDeep2 file/Know_Structure/LP42A2/oar-mir-27a.pdf]

miRBase precursor : oar-mir-27a
Total read count : 308097
oar-miR-27a read count : 307858
remaining reads : 239

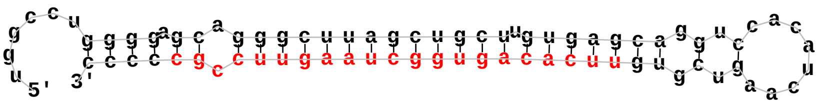

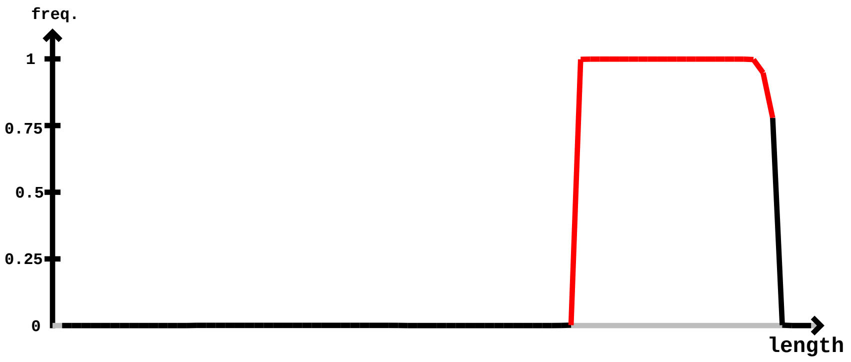


```
                                                    oar-miR-27a
5'- uggccuggggagcagggcuuagcugcuugugagcagguccacaucaagucguguucacaguggcuaaguuccgccccc  -3'    exp
    ......((((.((.(((((((((((((.(((((((.(.(........).).))))))))))))))))))))).))))))  reads      mm        sample
    .............agggcuuagcugcuugug...............................................  1          0         seq
    .............agggcuuagcugcuugugag.............................................  2          0         seq
    .............agggcuuagcugcuugugagc............................................  46         0         seq
    .............agggcuuagcugcuugugagca...........................................  181        0         seq
    .............agggcuuagcugcuugugagcag..........................................  2          0         seq
    .............agggcuuagcugcuugugagcagg.........................................  6          0         seq
    ..............gggcuuagcugcuugugagca...........................................  1          0         seq
    ...................................................uguucacaguggcuaagu.........  3          0         seq
    ...................................................uguucacaguggcuaaguuc.......  2          0         seq
    ...................................................uguucacaguggcuaaguucc......  10         0         seq
    ...................................................uguucacaguggcuaaguucU......  5          1         seq
    ...................................................uguucacaguggcuaaguuccg.....  83         0         seq
    ...................................................uguucacaguggcuaaguuccgc....  13         0         seq
    ...................................................uguucacaguggcuaaguuccgcc...  2          0         seq
    ....................................................guucacaguggcuaaguucc......  2          0         seq
    ....................................................guucacaguggcuaaguucU......  11         1         seq
    ....................................................guucacaguggcuaaguuccg.....  1          0         seq
    ....................................................guucacaguggcuaaguucUg.....  58         1         seq
    ....................................................guucacaguggcuaaguucUgc....  308        1         seq
    ....................................................guucacaguggcuaaguuccgc....  3          0         seq
    ....................................................guucacaguggcuaaguuccgcc...  1          0         seq
    .....................................................uucacaguggcuaaguu........  40         0         seq
    .....................................................uucacaguggcuaagCu........  1          1         seq
    .....................................................uucacaguggcuaaguuG.......  3          1         seq
    .....................................................uucacaguggcuaaguuc.......  509        0         seq
    .....................................................uucacagugUcuaaguuc.......  1          1         seq
    .....................................................uucacaguggcuaaguucc......  2769       0         seq
    .....................................................uucacaguggcuaaguucU......  12418      1         seq
    .....................................................uucacaguggcuaaguucUg.....  26111      1         seq
    .....................................................uucacaguggcuaaguuccg.....  19798      0         seq
    .....................................................uucacaguggcuaaguuccA.....  6098       1         seq
    .....................................................uucacaguggcuaaguucUgc....  232677     1         seq
    .....................................................uucacaguggcuaaguuccgc....  6151       0         seq
    .....................................................uucacaguggcuaaguuccgcc...  493        0         seq
    .....................................................uucacaguggcuaaguuccgccc..  3          0         seq
```

oar-miR-27a

uggccuggggagcagggcuuagcugcuugugagcagguccacaucaagucguguucacaguggcuaaguuccgccccc			
....ucacaguggcuaaguucc......	1	0	seq
....ucacaguggcuaaguucU......	8	1	seq
....ucacaguggcuaaguuccA.....	2	1	seq
....ucacaguggcuaaguuccg.....	12	0	seq
....ucacaguggcuaaguucUg.....	13	1	seq
....ucacaguggcuaaguucUgc....	211	1	seq
....ucacaguggcuaaguuccgc....	6	0	seq
....cacaguggcuaaguucU......	1	1	seq
....cacaguggcuaaguuccg.....	2	0	seq
....cacaguggcuaaguuccA.....	1	1	seq
....cacaguggcuaaguucUgc....	22	1	seq
....acaguggcuaaguucUg.....	1	1	seq
....acaguggcuaaguucUgc....	4	1	seq
